# Pulmonary Function Tests: Easy Interpretation in Three Steps

**DOI:** 10.3390/jcm13133655

**Published:** 2024-06-22

**Authors:** Josuel Ora, Federica Maria Giorgino, Federica Roberta Bettin, Mariachiara Gabriele, Paola Rogliani

**Affiliations:** 1 Division of Respiratory Medicine, University Hospital Tor Vergata, 00133 Rome, Italy; 2 Unit of Respiratory Medicine, Department of Experimental Medicine, University of Rome “Tor Vergata”, 00133 Rome, Italy; 3 Department of Emergency Medicine, Fondazione Policlinico Tor Vergata, Viale Oxford 81, 00133 Rome, Italy

**Keywords:** spirometry, lung volumes, interpretation, PRISm, dysanaptic patterns

## Abstract

Pulmonary function tests (PFTs) are pivotal in diagnosing and managing a broad spectrum of respiratory disorders. These tests provide critical insights into lung health, guiding diagnoses, assessing disease severity, and shaping patient management strategies. This review addresses the complexities and nuances inherent in interpreting PFT data, particularly in light of recent updates from the European Respiratory Society (ERS) and American Thoracic Society (ATS). These updates have refined interpretive strategies, moving away from definitive diagnostic uses of spirometry to a more probabilistic approach that better accounts for individual variability through the use of Z-scores and lower limits of normal (LLNs). Significantly, this narrative review delves into the philosophical shift in spirometry interpretation, highlighting the transition from direct clinical diagnostics to a more nuanced evaluation geared towards determining the likelihood of disease. It critiques the reliance on fixed ratios and emphasizes the need for reference values that consider demographic variables such as age, sex, height, and ethnicity, in line with the latest Global Lung Function Initiative (GLI) equations. Despite these advances, challenges remain in ensuring uniformity across different predictive models and reference equations, which can affect the accuracy and consistency of interpretations. This paper proposes a streamlined three-step framework for interpreting PFTs, aiming to unify and simplify the process to enhance clarity and reliability across various medical specialties. This approach not only aids in accurate patient assessments but also mitigates the potential for misdiagnosis and ensures more effective patient management. By synthesizing contemporary guidelines and integrating robust physiological principles, this review fosters a standardized yet flexible approach to PFT interpretation that is both scientifically sound and practically feasible.

## 1. Introduction

Pulmonary function tests (PFTs) are crucial tools in the field of respiratory medicine, allowing medical practitioners to assess lung function and helping to diagnose a wide range of respiratory disorders. These tests provide insights into various aspects of lung health, playing a pivotal role in diagnosis, assessing disease severity, and guiding ongoing patient management. However, the data yielded by PFTs can be intricate, especially for those not intimately familiar with their interpretation.

The ERS and ATS recently revised their interpretive strategies for routine PFTs [1], alongside their established spirometry standardization [2], building upon prior standardization efforts [3,4]. While this narrative review primarily focuses on the interpretive aspects rather than technical standards, it is essential to underscore the importance of sound technical practices. A robust technique is fundamental for accurate interpretation.

Although this review will not delve deeply into technical evaluations, certain prerequisites must be met to ensure the validity of the data under analysis. These prerequisites include verifying anthropometric measurements, confirming spirometry contraindications, and, critically, assessing acceptability and reproducibility [2]. While it is theoretically possible to interpret any data, adherence to acceptability and reproducibility criteria significantly enhances the interpretation’s fidelity to actual physiological conditions.

Some foundational principles need to be established to better grasp the philosophy behind the new interpretation of spirometry. Perhaps the most significant shift lies in recognizing spirometry as a tool more adept at gauging the likelihood of disease rather than serving as a definitive diagnostic tool. This paradigmatic shift marks a substantial departure from the interpretative strategies of 2005 to those of 2021 [1,4].

Like the majority of physiological parameters, respiratory measures adhere to a normal Gaussian distribution. Consequently, determining whether a specific parameter falls within the realm of normalcy or deviates into pathology can be a nuanced challenge. The farther a parameter deviates from the median, the greater the likelihood of pathology. In contemporary practice, the Z-score, precisely quantifying how a value deviates from the median, has supplanted the use of the percentage of predicted (%pr) approach. This transition underscores the importance of a more refined understanding of individual variation and its implications in spirometric interpretation.

The LLNs do not necessarily indicate a pathophysiological abnormality, nor do they serve as a clinically meaningful threshold for diagnosing diseases. Instead, they offer insight into whether the observed result aligns with what can be expected in otherwise healthy individuals of similar age, sex, and height (for more details, see Ref. [1]). This signifies a departure from the notion, clearly stated in the 2005 strategies, that pulmonary function tests can be used for direct clinical diagnosis [3]. For this reason, in this review, the term “ventilatory defect” has been replaced with ‘spirometric pattern’, as was the case in previous papers [5,6]. This change underscores that spirometry results should not be viewed as a standalone clinical diagnosis but must be interpreted in conjunction with other preclinical tests and clinical symptoms [7]. Additionally, it is important to note that normal spirometric results do not necessarily indicate the absence of underlying lung issues. Other tests, such as the forced oscillometry technique (FOT) and cardiopulmonary exercise test (CPET), among others, may be needed to detect potential problems [8].

A “ventilatory defect” refers to an abnormality in the process of ventilation, which depends on the mechanical properties of the respiratory system, the conditions under which the subject is tested (rest, exercise, or sleep), and the test used (spirometry, CPET, FOT, etc.). Traditionally, there are two main types of ventilatory defects: obstructive spirometric pattern, commonly caused by chronic obstructive pulmonary disease (COPD), asthma, and bronchitis, characterized by symptoms such as dyspnea (shortness of breath), chronic cough, and wheezing [9]; and restrictive pattern, caused by interstitial lung disease, chest wall deformities, and neuromuscular conditions, characterized by exertional dyspnea, fatigue, dry cough, and reduced exercise tolerance [10]. On the other hand, the “spirometric pattern” specifically relates to the results obtained from spirometry.

**Figure 1:**
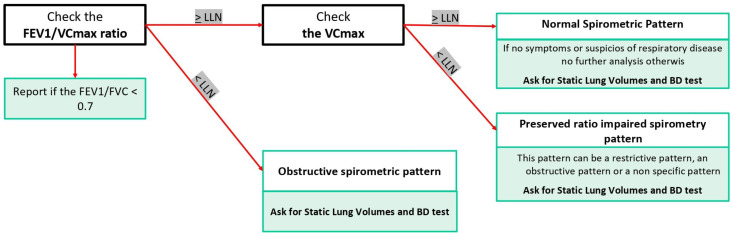
Forced spirometry simplifies interpretation. The figure illustrates an intuitive method for interpreting forced spirometry results. Commencing with the FEV1/VCmax ratio, if it falls below the LLN, it indicates an obstructive pattern; otherwise, attention shifts to the FVC value. A normal FVC suggests a normal spirometric pattern, whereas a reduced FVC may indicate a PRISm. Report if the FEV1/VCmax is lower than 0.7. BD: bronchodilator; FEV1: forced expiratory volume in one second; FVC: forced vital capacity; LLN: lower limit of normal; PRISm: preserved ratio impaired spirometry.

**Figure 2:**
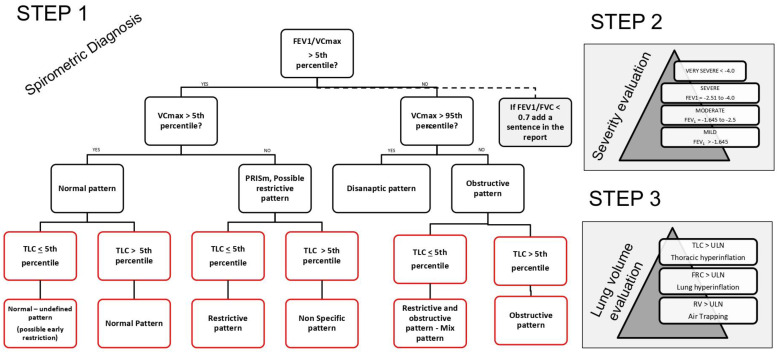
Unified Flow Chart (UFC): Three-Step Interpretation of Spirometry and Lung Volumes. This flow chart provides a unified approach to interpreting spirometry (black boxes) and lung volumes (red boxes), encompassing three key steps. By following this flow chart, one can effectively interpret spirometry results alongside lung volumes, assess disease severity based on FEV1, and define hyperinflation. Step 1: Interpret Spirometry with Lung Volumes; Step 2: Assess Disease Severity Based on FEV1; Step 3: Define Hyperinflation. BD: bronchodilator; FEV1: forced expiratory volume in one second; FRC: functional residual capacity; FVC: forced vital capacity; LLN: lower limit of normal; PRISm: preserved ratio impaired spirometry; RV: residual volume; TLC: total lung capacity; ULN: upper limit of normal; VCmax: maximal vital capacity.

Although interpretation of spirometry and diagnosis are two distinct aspects of the same coin, it is also true that certain clinical recommendations, such as those from GOLD, and findings from clinical trials have significantly influenced the reporting approach. Hence, even though a fixed forced expiratory volume in one second (FEV1)/forced vital capacity (FVC) ratio post-bronchodilator (BD) lower than 0.7 was never explicitly recommended in spirometry guidelines, it is frequently employed as a marker of an “obstructive pattern” following the GOLD recommendation. The objective is to reconcile this contrast in the unified flow chart (UFC) through the integration of these two perspectives (Figure 1 and Figure 2).

Interpreting pulmonary function tests presents the challenge of needing reference values that consider factors such as sex, age, height, and ethnicity. Over time, multiple reference equations have emerged. In 2012, the Global Lung Function Initiative (GLI) published the latest reference equations for spirometry [11], drawing from data collected from over 97,000 individuals of diverse ethnic backgrounds, ranging from 3 to 95 years of age. In 2017, GLI released reference equations for gas diffusion capacity for carbon monoxide (DLCO) based on data from more than 12,000 individuals [12] and, in 2021, they introduced reference equations for static lung volumes, derived from tests on 7000 individuals aged 5 to 85/80 years old [13].

The GLI equations are now the recommended choice for reference values [14]. However, this approach, while currently predominant, does have certain limitations. Firstly, it lacks predicted values for all possible combinations of age, gender, and ethnicity [14]. Additionally, because all results are relative to the predicted values, the interpretation of severity can vary depending on the chosen predicted values. This variability can pose challenges when comparing studies that use different reference equations [15,16]. Furthermore, when comparing a fixed ratio to an LLN ratio, the fixed-ratio approach appears to be more robust [17].

While it is true that every approach has its critiques, this review embarks on a mission to offer a simple yet not oversimplified method for interpreting PFTs that can be embraced by physicians across specialties. Simplicity and practicality stand as the pillars for unifying PFT interpretation. Strong physiological foundations serve as the cornerstone for comprehending interpretations and applying them to individual cases.

Interpreting PFT results extends beyond an intellectual exercise; it profoundly impacts patient care. An erroneous interpretation can result in incorrect diagnoses, suboptimal treatment plans, and compromised patient well-being. Hence the pressing need for a systematic and accessible approach to deciphering PFT findings. In response to this need, this paper introduces a concise three-step framework designed to streamline the interpretation process, facilitating precise diagnoses and informed clinical decisions.

There are two points not addressed in this review: bronchodilation and DLco. The guidelines for interpreting bronchodilation results have recently changed according to spirometric guidelines, now evaluating a positive response if the FEV1 or FVC increases by more than 10% of the predicted value. However, both GINA [18] and GOLD [9] have not adopted this change and continue to use the previous criteria (FEV1 or FVC greater than 12% of the baseline and 200 mL). Conversely, the interpretation of DLco remains unchanged, with only the severity now being assessed using Z-score criteria [1,19]. For further understanding of DLco or DLco/VA, please refer to the following papers [20].

### 1.1. Before Starting

Before delving into the numerical data and interpreting the results of PFTs, it is essential to first evaluate the quality of the spirometry maneuvers. The cornerstone of accurate PFT interpretation lies in the acceptability and reproducibility of these maneuvers [2,4]. To ensure high-quality reproducibility, at least three acceptable maneuvers are required, though two may suffice for acceptable quality under certain conditions. It is crucial to consider both safety—ensuring there are no contraindications to performing the test—and quality, which encompasses acceptability and reproducibility of the maneuvers. The usability of the data may be deemed sufficient in some cases, but always verify whether pre-test bronchodilator therapy has been administered, as this can significantly affect the test outcomes. Prioritizing these aspects sets a solid foundation for accurate interpretation of PFT results.

### 1.2. First Step: Interpreting Spirometry Results

#### 1.2.1. Forced Spirometry: Flow Volume Curve

The force–volume curve is undoubtedly the most commonly employed test. The principal parameters for interpretation include FVC, FEV1, and the FEV1/FVC ratio. While numerous other parameters are observable, thus far, none have proven sufficiently important or reproducible to be considered in the initial evaluation [21]. Consequently, recommendations do not prioritize these additional parameters as a first step.

Interpretation of spirometry commences with the FEV1/FVC ratio (Figure 1). If this ratio is above the LLN, indicating normalcy, the focus then shifts to the FVC. Here, two scenarios emerge: if FVC is normal, the spirometric pattern is deemed normal; if FVC is below the LLN, a preserved ratio impaired spirometry pattern (PRISm pattern) is identified. Conversely, if the FEV1/FVC ratio is below the LLN, it bifurcates into either an obstructive pattern, if FVC is normal, or an obstructive/mixed pattern, should FVC also be below normal (Figure 1).

It is essential to recognize that the flow–volume curve is primarily diagnostic of obstructive spirometric patterns, with other patterns potentially presenting ambiguities. For instance, a normal pattern could misleadingly indicate a restrictive pattern if the TLC is below the LLN. Similarly, a PRISm pattern could represent an obstructive, non-specific, or restrictive pattern if TLC data are unavailable.

A normal pattern, characterized by an FEV1/FVC ratio within the normal range and an FVC exceeding the LLN, poses questions about its predictive value for normality. It is important to note that, according to recommendations, the restrictive pattern is defined based on TLC rather than FVC alone, given that a normal FVC does not preclude a decreased TLC. Aaron et al. have shown that a normal FVC has a negative predictive value below 3% for predicting a restrictive pattern when defined by total lung capacity [22]. Nevertheless, there remains a small chance of false negatives, underscoring that if clinicians suspect interstitial disease, evaluating static lung volumes is still warranted. Hence, in Figure 1, we indicate that absent symptoms or pre-test probability of restrictive disease, the spirometric pattern is considered normal, and the test may conclude at this juncture.

Moreover, the initial analysis of the FEV1/FVC ratio raises two issues: whether to use VC or FVC and whether to apply the fixed ratio or LLN. Previously, VCmax (i.e., the highest VC from SVC and FVC) was preferred over FVC in the ratio [3], and current authors compiling recommendations acknowledge its greater sensitivity compared to the FEV1/FVC ratio, despite its lesser specificity [23]. There are also measurement challenges associated with VC [24], and no GLI reference values exist for the VC and FEV1/VC ratio. Opting for the previously recommended FEV1/VC ration to diagnose airflow obstruction introduces uncertainty, especially in older populations [1], whereas using FVC instead of VCmax might underestimate the diagnosis of an obstructive pattern [25]. An SVC significantly exceeding the FVC (>100 mL) indicates airway collapse during forced exhalation [26], a sign of obstruction and small airways disease [21,27]. Furthermore, a normal FEV1/FVC ratio does not assure the absence of flow limitation or small airways disease, as FOT can reveal impedance changes despite normal FEV1/FVC values [28]. Returning to historical methodologies, the true Pinelli-Tiffeneau index is defined as the FEV1/VC ratio [29]. This metric is favored due to its superior assessment of static lung volumes through a quasi-static maneuver, such as the slow vital capacity (SVC), in contrast to forced maneuvers that may exacerbate alveolar collapse [27,30], resulting in lower measurements. For these reasons, SVC should be preferred. Alternatively, integrating the differences, the maximum vital capacity (VC max) could be a valid option, as we utilized in the UFC. As previously mentioned, this index (FEV1/VCmax) could overestimate obstruction in elderly subjects, so it is always important to integrate the spirometric pattern with the patient’s symptoms.

The ongoing debate between the LLN and fixed ratio for FEV1/FVC [31,32] reflects the challenges of diagnosing conditions like COPD, particularly in the elderly. The LLN accounts for age-related physiological changes [20], whereas the fixed ratio offers simplicity for clinical use. Yet, this debate continues, with recent studies and recommendations presenting divergent views on the most effective approach of COPD [17,33].

Addressing these challenges (Figure 2), the unified flow chart (UFC) incorporates FEV1/VCmax to enhance the sensitivity of detecting obstructive patterns. It strongly recommends reporting if the FEV1/FVC is below 0.7, allowing for the possibility of identifying a normal spirometric pattern in the elderly with a reduced ratio. This approach alleviates potential issues regardless of whether only the forced maneuver or both slow and forced maneuvers are performed, using VCmax. Additionally, indicating if the FEV1/FVC ratio is below 0.7 provides a clearer pathway to diagnosing COPD, assuming other conditions are met.

Physicians should be aware that, ultimately, the GOLD criteria tend to over-diagnose COPD, whereas LLN definitions under-diagnose it in elderly patients as compared to diagnoses by expert panels [34], particularly in elderly males. LLN is more accurate in diagnosing early COPD in individuals younger than 44 years [35]. Therefore, clinical evaluations and diagnoses rest with the physician, rather than solely relying on the flow–volume curve maneuver, which only indicates the spirometric pattern

#### 1.2.2. Preserved Ratio Impaired Spirometry (PRISm)

During spirometry, one may observe diminished values in both FEV1 and FVC, yet with a preserved ratio. Some authors have suggested this as a potential restrictive pattern, emphasizing the need for total lung capacity (TLC) assessment to draw definitive conclusions. Others categorize it as a ‘non-specific’ spirometric pattern, but the term ‘Preserved Ratio Impaired Spirometry’ (PRISm) has gained recent popularity [1,36,37,38].

In 2014, Wan et al. first coined the term PRISm, stating, “Approximately 1 out of every 8 subjects in the general population exhibits PRISm, alternatively referred to as ‘unclassified’, ‘non-specific’, or ‘restrictive’ spirometry, with the latter term being the most widely accepted” [39]. Guerra et al. had previously identified the PRISm pattern, terming it a “restrictive spirometric pattern” in 12% of 2048 participants [5]. Additionally, their findings linked both consistent and inconsistent restrictive spirometry patterns to a heightened risk of mortality, introducing the concepts of PRISm trajectory and associations between various impaired spirometric patterns and increased mortality risk.

Presently, much like other aspects of spirometry, a notable incongruence exists between recommendations and clinical studies, and PRISm is no exception. While current guidelines categorize PRISm alongside other physiological impairments based on the lower limit of normality, defining it as a normal FEV1/FVC ratio (greater than LLN) and an FVC lower than LLN [1]; the literature commonly adopts an operational definition of FEV1/FVC greater than 0.70 and FEV1 lower than 80% predicted [37,39]. Similarly, the GOLD recommendation defines the patterns PRISm or preCOPD as an FEV1/FVC greater than 0.70 and an FEV1 lower than 80% predicted post bronchodilation. It is important to highlight that in numerous studies exploring this pattern, particularly epidemiological studies focused on COPD, there is a tendency to include FEV1 (<80%pr) rather than FVC. This inclination stems from the context of COPD research, where FEV1 carries more significance and is more commonly used than FVC [36,39,40]. Depending on the primary focus of the study, which may involve identifying restrictive or obstructive diseases, PRISm can be defined by either a lower FVC or lower FEV1 [41,42] while maintaining a normal FEV1/FVC ratio.

A subtle aspect arises when considering that, in the recommendation flow chart, this pattern is labeled as a possible restrictive or non-specific pattern [1]. Conversely, in the table, the authors designate a non-specific pattern only when the TLC is within normal limits, implying that a non-specific pattern should align with a PRISm pattern possessing a normal TLC [43]. The distinction lies in the fact that the PRISm pattern, within the general population, likely represents a restrictive spirometric pattern (when integrated with TLC) or an obstructive pattern in high-risk populations such as smokers (preCOPD). On the other hand, a non-specific pattern (PRISm with TLC) presents a more intricate impairment that is challenging to interpret [6,44].

The decline in vital capacity (VC) in PRISm is likely attributable to a combination of factors, with varying influences from high body mass index (BMI), tobacco smoking, advanced age, female sex, anatomically small lungs, concurrent restrictive abnormalities, pulmonary gas trapping, impaired lung development, diabetes, and cardiovascular disease [6,45,46,47,48]. In any case, PRISm has been linked to heightened mortality risks, as highlighted by a recent meta-analysis conducted by Yan et al. [49]. This comprehensive study, encompassing eight clinical studies and involving 40,699 individuals diagnosed with PRISm, consistently demonstrated a significant association between PRISm and increased risks across various mortality categories. Furthermore, recent studies focused on COPD populations have demonstrated that PRISm is autonomously linked to chronic dyspnea, exercise limitations, diminished quality of life, and even heightened mortality, with a prevalence ranging from 3% to 20% [45,50]. Despite these findings, the underlying pathophysiological mechanisms contributing to increased dyspnea and decreased exercise capacity in individuals with preserved FEV1/FVC (i.e., PRISm) and the potential intersections with low fixed-ratio COPD remain largely unexplored [9,44,51].

In conclusion, the PRISM pattern may be classified as obstructive, restrictive, or a nonspecific pattern (NSP). Regardless of the underlying cause, this pattern is associated with an increased risk of mortality and dyspnea, warranting further investigation. Therefore, it is crucial to conduct additional tests, including lung volume and bronchodilation assessments, to explore this association more thoroughly Figure 2.

#### 1.2.3. Dysanaptic Pattern

The dysanaptic pattern identified in spirometry highlights an imbalanced growth of airways compared to lung volume, which has important consequences for pulmonary function [52,53]. In clinical settings, including screening, it is often observed that patients exhibit airflow limitation on spirometry despite having supernormal percent-predicted FEV1 and FVC values. These individuals are typically healthy non-smokers without airway symptoms or a prior diagnosis of pulmonary disease. This scenario complicates the accurate identification of their pathophysiological states and may lead to overdiagnosis of obstructive pulmonary disease. It should be noted that larger lung size does not necessarily correlate with larger airways [54].

This pattern holds particular significance in pediatric populations undergoing lung development [55,56] and in athletes, who may exhibit larger lung volumes [57,58]. The term “dysanaptic” suggests that the increase in airway size does not keep pace with lung volume, potentially resulting in functional restrictions and a heightened risk of respiratory conditions.

Spirometry is a critical tool for detecting the dysanaptic pattern by measuring the amount of air an individual can exhale and the velocity of exhalation following a maximal inhalation [54]. Essential metrics, including FVC and FEV1, are pivotal for diagnosing airflow obstruction and evaluating lung development. Additionally, some studies have considered the ratio of forced expiratory flow between 25% and 75% of lung volume over FVC (FEF25-75/FVC) [59].

The dysanaptic pattern is characterized by an FEV1/FVC ratio below the LLN with both FEV1 and FVC exceeding LLN, signifying normal FEV1 and FVC levels [1]. According to updated guidelines, this condition signifies a convergence of dysanaptic growth and mild obstruction. Nonetheless, some researchers point out the resemblance between dysanapsis and pre-obstruction, suggesting potential interchangeability [60,61].

The critical issue in assessing a subject involves determining whether the dysanapsis pattern represents a preclinical stage of chronic obstructive pulmonary disease (COPD) or merely an imbalanced growth between the airways and the parenchyma. Due to errors in classifying severity in recent guidelines [1], dysanapsis may be identified in cases where the FEV1/FVC ratio is below the LLN and FEV1 remains normal, rather than being classified as mild obstruction. However, the UFC guidelines recommend defining dysanapsis specifically for subjects with an obstructed FEV1/FVC ratio (<LLN) and an FVC greater than the upper limit of normal (ULN) (Figure 2). This distinction emphasizes the considerable disparity between lung capacity (measured by FVC) and airflow rate (measured by FEV1). Monitoring these subjects over time, along with a comprehensive medical history, is essential for a more accurate understanding.

#### 1.2.4. Interpreting Static Lung Volume Measurements

For a complete interpretation of spirometry, it is essential to evaluate static lung volumes such as total lung capacity (TLC), residual volume (RV), and functional residual capacity (FRC). Each of these volumes provides valuable insights into the health and efficiency of the respiratory system. To facilitate easy interpretation, the most crucial volume is TLC, which indicates whether the lungs are smaller than normal, identifying a restrictive pattern, or within the normal range or greater, which is compatible with normal lungs or an obstructive pattern.

A necessary premise is that, despite the recommendations not differing significantly on measurement methods, there are at least three traditional methods for measuring lung volumes, each with some differences: (1) plethysmography (body box), (2) gas dilution, which includes helium dilution and nitrogen washout, and (3) the recently introduced minibox [62]. Additionally, optoelectronic plethysmography and CT scans are used primarily for research in estimating lung volumes [63].

Plethysmography measures lung volumes by assessing the pressure changes in a closed system (body box) as the patient breathes. This method is highly accurate for measuring lung volumes difficult to assess with other methods, such as RV and TLC [64]. It is particularly useful in patients with severe obstructive lung diseases where gas distribution may be uneven. However, the equipment is bulky and expensive, limiting its availability in some settings, and requires active patient cooperation, which may be challenging for some individuals.

Gas dilution techniques, including helium dilution and nitrogen washout, measure lung volumes by having the patient breathe in and mix either helium or 100% oxygen with the air in their lungs. Although there are some differences between them, both methods are simpler than plethysmography but less accurate for patients with significant airflow obstruction. They tend to underestimate TLC and take a longer time to reach equilibrium, making them less reproducible in real life due to the limited number of maneuvers allowed [65].

Based on the latest recommendations [1], the interpretation of lung volumes begins by assessing total lung capacity (TLC). If TLC is found to be normal, exceeds the ULN, or falls below the LLN, the evaluation can indicate a range of possibilities from normal lung function to conditions like large lung volumes, hyperinflation, or various respiratory disorders. The framework introduces two specific indices at the same decision point in the flowchart: the RV/TLC and FRC/TLC ratios. This integration aims to refine diagnostic accuracy. However, the flowchart also introduces the FEV1/FVC ratio inconsistently, appearing in one section but absent in others, which complicates the interpretation and has been noted to introduce contradictions and complexities, as highlighted in other critiques [43].

The 2005 flowchart was more straightforward and easier to interpret compared to the latest version [3]. Its primary limitation was the inclusion of DLCO, which, while useful for clinical diagnostics, is beyond the intended scope of spirometric interpretation.

In an effort to balance clarity and comprehensive analysis, the UFC merges elements from both the old and new flowcharts. It maintains the simplicity of the earlier version while adopting the probabilistic approach of the recent guidelines. This merger avoids the confusion of integrating multiple indices such as FRC/TLC and RV/TLC directly into the main flowchart. These indices are considered important but are discussed separately to keep the primary diagnostic pathway clear (as illustrated in Figure 2). TLC assessments follow spirometric evaluations in clinical practice, and the DLCO is omitted from the UFC as it is pivotal in assessing pulmonary gas exchange rather than in the interpretation of dynamic and static lung volumes.

According to the UFC (Figure 2), there is a scenario where the spirometric pattern appears normal after measuring static lung volumes, which can remain normal or present a normal/undefined pattern. This represents a rare case where dynamic volumes (FEV1 and FVC) are normal, but the total lung capacity (TLC) is reduced due to a decreased residual volume (RV). According to the latest recommendations, this situation would present a paradox: using only forced spirometry, the pattern would be reported as normal, yet following the measurement of TLC, it would be classified as restrictive. To address this issue, we have chosen to categorize this case as normal/undefined (possible early restriction). This designation suggests that there might be an ongoing issue, but it remains unclear based on spirometry alone. This case could arise from a measurement error of the FRC/RV by the technique used, represent an early stage of a restrictive condition, or merely be an undefined pattern that falls within the normal variability.

After lung volume measurements, the PRISm pattern may evolve into a restrictive pattern or a nonspecific pattern when the TLC is greater than the LLN. In such cases, a bronchodilation test is necessary. If positive, this pattern equates to an obstructive pattern; otherwise, it remains a nonspecific pattern, essentially a PRISm pattern with normal TLC. It is important to note that a true PRISm pattern is identified when TLC data is not available.

Conversely, if forced spirometry results in an obstructive pattern, measuring TLC is crucial to exclude mixed disorders characterized by an FEV1/FVC ratio less than LLN and a TLC less than LLN.

Regarding the evaluation of lung volumes, which is beneficial for assessing hyperinflation (refer to step 3 in Figure 2), it is essential to evaluate each lung volume individually. If any volume is greater than normal (greater than ULN), diagnoses such as thoracic hyperinflation (TLC), lung hyperinflation (FRC), and air trapping (RV) can be made. The only exception occurs when spirometry results are normal, indicating that these might simply be normal or large volumes. In this particular scenario, the FRC/TLC or RV/TLC ratio is very useful for solving this problem.

**Figure 3:**
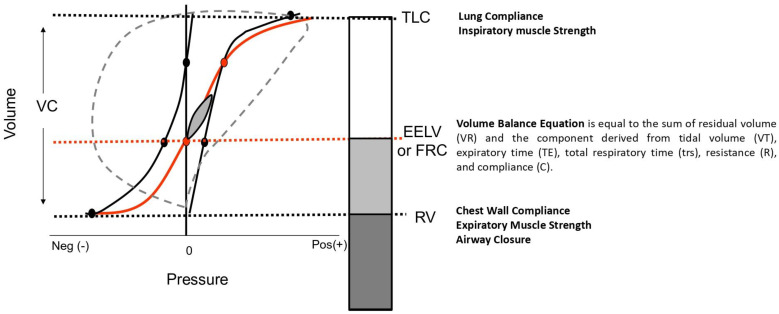
Pressure–Volume Curve and Volume Determinants. This figure illustrates the relationship between lung volumes and their determinants. It provides a visual representation of TLC, EELV or FRC, and RV. TLC: total lung capacity; EELV: end-expiratory lung volume; FRC: functional residual capacity; RV: residual volume.

While spirometric diagnosis may seem straightforward, interpreting and understanding lung mechanics is more complex. Physiology can aid in the interpretation of lung volumes. Briefly (as depicted in Figure 3), each lung volume has its determinants, and a deep understanding of both static and dynamic determinants can assist physicians in interpreting the physiological mechanisms underlying these measurements [20].

Various conditions can affect the properties of the respiratory system in multiple ways, altering the elastance/compliance of the lung or chest wall, or impacting the closing volume or time of emptying. In any case, lung functions will be affected. For example, in mild to moderate obesity, higher lung–chest wall elastic recoil may increase expiratory flows [20]. FRC decreases exponentially in the early stages of obesity; consequently, ERV decreases, while IC increases in tandem with BMI. RV and TLC may change only in very severe obesity. FVC frequently leads to underestimation of the relatively preserved SVC as the small airways might be compressed or collapse at the end of forced expiration. Therefore, in obese subjects, a normal or restrictive pattern can typically be observed, characterized by a decreased ERV [1]. Although the observed pattern may suggest obesity, the diagnosis of the pattern and the underlying pathology must be considered distinct, as many diseases can present overlapping patterns. For instance, PRISm is an example of such overlap.

### 1.3. Step 2 and Step 3

Following the identification of a spirometric pattern, the next steps involve assessing severity and defining lung volumes. Severity is determined primarily based on the forced expiratory volume in one second (FEV1), which reflects the respiratory system’s functional capacity. A compromised FEV1 indicates potential respiratory system alteration or disease [66]. Classifying the severity of respiratory conditions based on lung volumes other than FEV1 frequently results in contradictions, as evidenced by recent guidelines [43]. Specifically, applying a ‘mild’ classification to normal FEV1 values in an obstructive pattern introduces inconsistencies. Similarly, it is paradoxical to label a restrictive disease as ‘mild’ when total lung capacity (TLC) is normal. This latter scenario is inherently contradictory because a characteristic feature of restrictive patterns is reduced TLC. Hence, relying on FEV1 as the primary measure for determining severity avoids these inconsistencies and provides a clearer, more consistent framework for interpreting spirometric results.

FEV1 should be considered a valuable indicator for classifying the severity of respiratory disorders [67,68]. In terms of quantifying this severity, the FEV1 is interpreted as follows (Figure 2 step 2): a pattern is considered ‘mild’ if FEV1 is between −1.645 and −2.5 standard deviations from the norm, ‘severe’ if between −2.5 and −4, and ‘very severe’ if below −4 standard deviations.

Step three of the evaluation process focuses on assessing static lung volumes, which are crucial for diagnosing conditions like lung hyperinflation (Figure 2 step 3). Integrating clinical trials, which traditionally use percent predicted values, with the newer method of reporting based on Z-scores presents challenges [69,70]. To harmonize old classifications with new parameters, three distinct possibilities are recognized: air trapping when residual volume (RV) exceeds the ULN, lung hyperinflation when FRC exceeds ULN, and thoracic hyperinflation when total lung capacity (TLC) exceeds ULN [71,72].

However, relying solely on these classifications can lead to underestimating subjects with restrictive diseases or overestimating healthy individuals based on lung volumes alone. Therefore, using ratios like RV/TLC or FRC/TLC is essential to determine whether variations in lung volumes are pathological [1]. These ratios provide additional insight into the relationship between different lung capacities and help identify if observed abnormalities are clinically significant.

### 1.4. How to Write the Report of the Spirometry and Clinical Cases

**Figure 4:**
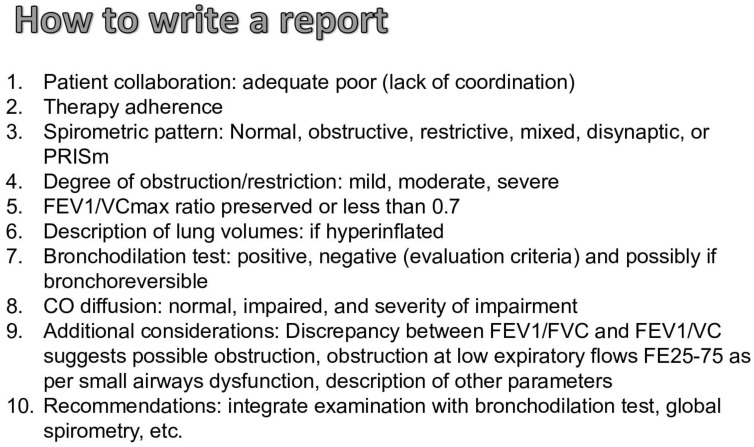
Steps for Preparing Spirometric Reports. This figure outlines the sequential steps involved in creating a comprehensive spirometric report. It provides a visual guide to ensure the proper preparation of spirometry reports.

When drafting a spirometry report, several critical elements must be systematically addressed to provide a comprehensive overview of the respiratory status. The following points should be considered (Figure 4).

Patient Collaboration: The report should begin by noting the quality of patient collaboration, categorized as either adequate or poor, the latter often indicated by a lack of coordination during the test. This factor is crucial for interpreting the reliability of the results.

Therapy Adherence: The adherence to prescribed respiratory therapies should be assessed and documented, as it can significantly influence the spirometric outcomes.

Spirometric Pattern: Identification of the spirometric pattern is essential. Patterns may include normal, obstructed, restricted, mixed, disanaptic, or PRISm. This classification helps guide further diagnostic and therapeutic decisions (see Figure 1 and Figure 2).

Degree of Obstruction/Restriction: The severity of any identified obstruction or restriction should be quantified as mild, moderate, or severe. This grading helps in assessing the progression of the disease and tailoring the management accordingly (Figure 2, step 2).

FEV1/VCmax Ratio: The ratio of forced expiratory volume in the first second to maximal vital capacity between the forced and the slow maneuvers, if both are available (FEV1/VCmax) should be noted, with specific attention to whether it is preserved or less than 0.7, which may suggest obstructive pathology (Figure 1).

Description of Lung Volumes: Any findings of hyperinflation should be explicitly mentioned as they are indicative of conditions such as emphysema or chronic obstructive pulmonary disease (Figure 2, step 3).

Bronchodilation Test: The results of bronchodilation testing should be recorded as positive or negative, based on established evaluation criteria. It is also beneficial to note if the condition is bronchoreversible [1].

CO Diffusion (if available): The diffusion capacity of carbon monoxide (CO) should be assessed and reported as normal, impaired, and the degree of impairment should be quantified to evaluate gas exchange efficiency, according to the z-score classification [1,20].

Additional Considerations: The report should include any discrepancies between the FEV1/FVC ratio and the FEV1/VC ratio, which might indicate potential obstruction. Observations of obstruction at low expiratory flows (e.g., FE25-75) could suggest small airway dysfunction. The inclusion of other relevant parameters is also advised, such as RV/TLC and FRC/TLC [20].

Recommendations: The report should conclude with recommendations for further testing, such as an integrated examination with a bronchodilation test and global spirometry to provide a more comprehensive evaluation of the respiratory system.

By meticulously documenting these aspects, the spirometry report will serve as a critical tool in diagnosing respiratory conditions, monitoring disease progression, and guiding treatment strategies.

**Figure 5:**
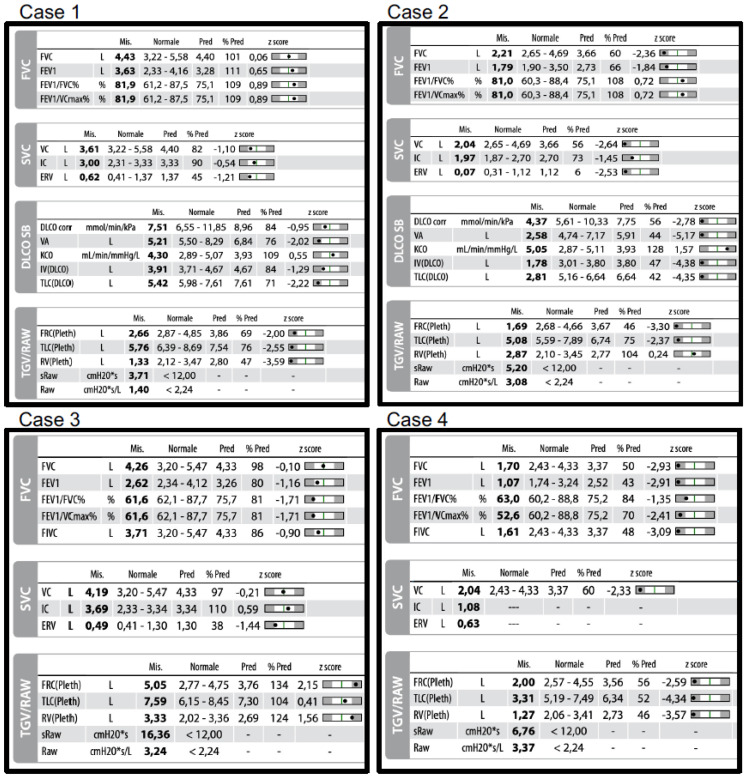
Four Spirometric Cases.

Four spirometric cases (Figure 5) will be presented. The characteristics of the subjects (age, sex, height) and their clinical diagnoses are not included to emphasize the use of z-scores in spirometric interpretation and to replicate what typically occurs in a functional test laboratory. The clinical interpretation, which involves considering many other factors, is beyond the scope of this review.

There are four different spirometric cases: Case 1: Normal—Undefined Pattern (possible early restriction); Case 2: Restrictive Pattern; Case 3: Obstructive Pattern with Lung Hyperinflation; and Case 4: Mixed Pattern (Restrictive and Obstructive). The explanation of the interpretation is provided in the main text (paragraph 4).

1. Case 1 (Figure 5)

Premise: Adequate coordination; the subject did not assume inhalatory therapy.
Step 1: Spirometry (Figure 1 and Figure 2)
  - FEV1/VC max is greater than the LLN (Z-score: 0.89) and the VC max is within the normal range (Z-score: 0.06), indicating normal spirometry.
  - Static lung volumes (plethysmography) (Figure 2): The TLC is lower than the LLN (Z-score: −2.55), suggesting a “Normal—undefined pattern (possible early restriction)”.

Step 2: Since the spirometry is normal, there is no classification of severity. However, if considered as a restrictive pattern, it would indicate mild restriction due to FEV1 being greater than the LLN (Z-score: 0.06).
Step 3: Lung Volume Evaluation
  - No lung or thoracic hyperinflation, no air trapping.

Other Tests:
  - Bronchodilation test not available.
  - DLco and DLco/VA are within the normal range, with a reduction in VA.
  - Observations: resistance is within the normal range.

Conclusions: Normal—undefined pattern. If clinically compatible, this could indicate possible early restriction.
Comments on this Spirometry: This is a challenging case of normal spirometry with impaired static lung volumes. According to spirometric guidelines, clinicians might have stopped the evaluation and reported this as normal based solely on slow/forced spirometry. However, if the patient undergoes static lung volume evaluation, the diagnosis should be revised to a restrictive ventilatory defect, contradicting the initial spirometry results [43]. According to recommendations, this case should be scored as mild based on the TLC, and normal based on the FVC. Therefore, it may be beneficial to standardize the diagnosis and severity criteria to ensure consistency.

2. Case 2 (Figure 5)

Premise: Adequate coordination; the subject did not assume inhalatory therapy.
Step 1: Spirometry (Figure 1 and Figure 2)
  - FEV1/VC max is greater than the LLN (Z-score: 0.72) and VC max is lower than the LLN (Z-score: −2.36), indicating a PRISm pattern.
  - Static lung volumes (plethysmography) (Figure 2): The TLC is lower than the LLN (Z-score: −2.37), suggesting a “restrictive pattern.”

Step 2: According to the evaluation of FEV1 (Z-score: −1.84), the severity of the restriction is moderate.
Step 3: Lung Volume Evaluation
  - No lung or thoracic hyperinflation, no air trapping.

Other Tests:
  - Bronchodilation test not available.
  - DLco is reduced, but DLco/VA is within the normal range, due to a reduction in VA.
  - Observations: Resistance is within the normal range. Despite a decrease in FRC and TLC, the RV is normal.

Conclusions: Moderate restrictive pattern with a severe decrease in the diffusion of CO, but a preserved DLco/VA. A reduction in DLco due to a decrease in alveolar volume is noted, but not in DLco/VA. Additionally, the residual volume is within normal limits, indicating air trapping in the context of a restrictive pattern.
Comments on this Spirometry: This is a classic case of a restrictive pattern. The differences between the UFC and the guidelines are as follows: the severity is evaluated according to FEV1; the score is different, as the UFC considers alterations between −1.645 and −2.5 as moderate, whereas spirometric recommendations classify this as mild. Additionally, because the RV/TLC ratio is increased, it should be reported as a complex restriction, but in the UFC, this is noted as a supplementary comment. According to Figure 1, if only the slow/forced maneuver had been performed, the report would have been: Pattern PRISm, possible restriction, with a recommendation to evaluate static lung volumes and perform a bronchodilation test.

3. Case 3 (Figure 5)

Premise: Adequate coordination; the subject assumed a bronchodilator (ICS/LABA) in the morning.

Step 1: Spirometry (Figure 1 and Figure 2)
  - FEV1/VC max is lower than the LLN (Z-score: −1.71) and VC max is within the normal range (Z-score: −0.10), indicating an obstructive pattern.
  - Static lung volumes (plethysmography) (Figure 2): The TLC is greater than the LLN (Z-score: 0.41), confirming an “obstructive pattern.”

Step 2: According to the evaluation of FEV1 (Z-score: −1.16), the severity of the obstruction is mild.
Step 3: Lung Volume Evaluation
  - TLC is within the normal range, the FRC is increased (Z-score: 2.15), and the RV is close to the ULN.

Other Tests:
  - Bronchodilation test and DLco not available.
  - Observations: Resistance is increased.

Conclusions: Mild obstructive pattern with lung hyperinflation. Additionally, the residual volume is close to the ULN, indicating a possible air trapping, and plethysmographic lung resistance is increased.
Comments on this Spirometry: This is a classic case of a mild obstructive pattern. The differences between the UFC and the guidelines are as follows: according to the recommendations, the severity of this case cannot be scored. This pattern could be easily interpreted as a dysanaptic pattern because the FEV1 and FVC are normal. To avoid this misinterpretation, we have arbitrarily defined the dysanaptic pattern as an obstructive pattern with an FVC greater than the ULN.

4. Case 4 (Figure 5)

Premise: Adequate coordination; the subject assumed a bronchodilator (ICS/LABA/LAMA) in the morning.

Step 1: Spirometry (Figure 1 and Figure 2)
  - FEV1/VC max is lower than the LLN (Z-score: −2.41) and VC max is lower than the normal range (Z-score: −2.93), indicating an obstructive pattern.
  - Static lung volumes (plethysmography) (Figure 2): The TLC is lower than the LLN (Z-score: −4.34), suggesting a “mixed restrictive and obstructive pattern.”

Step 2: According to the evaluation of FEV1 (Z-score: −2.91), the severity of the mixed pattern is severe.
Step 3: Lung Volume Evaluation
  - TLC, FRC, and RV are lower than the ULN.

Other Tests:
  - Bronchodilation test and DLco not available.
  - Observations: Resistance is within the normal range.

Conclusions: Severe mixed restrictive and obstructive pattern.
Comments on this Spirometry: This is a complex case of a mixed pattern. According to the guidelines, it should be reported as simply restrictive because the FEV1/FVC ratio is within the normal range, although close to the LLN. However, in this case, the FEV1/VC max is lower than the LLN, and the difference between VC and FVC is significant. Moreover, if only the forced spirometry was performed, this case would be reported as a PRISm pattern, but with the slow maneuver, it is identified as an obstructive pattern. This is a complex but illustrative case of how the two flowcharts differ.

## 2. Conclusions

Pulmonary function tests are indispensable for the elucidation and management of numerous respiratory diseases. This review has endeavored to streamline the interpretation of pulmonary function tests with the objective of facilitating standardized reporting practices. By harmonizing the disparate guidelines promulgated by recent recommendations, we foster the adoption of a universal nomenclature within the field. Despite these efforts, we strongly advocate for a physiologically oriented approach to PFT analysis as a subsequent phase. This approach, while recommended, carries the inherent risk of diverse interpretations across different centers, contingent on their specific expertise and experiential knowledge.

## Data Availability

Not applicable.

## Abbreviations

%pr	Percentage of Predicted
ATS	American Thoracic Society
BD	Bronchodilator
BMI	Body Mass Index
CPET	Cardiopulmonary Exercise Test
DLCO	Diffusing Capacity for Carbon Monoxide
ERS	European Respiratory Society
FEF25-75	Forced Expiratory Flow between 25% and 75% of lung volume
FEV1	Forced Expiratory Volume in one second
FOT	Forced Oscillometry Technique
FRC	Functional Residual Capacity
FVC	Forced Vital Capacity
GLI	Global Lung Function Initiative
GOLD	Global Initiative for Chronic Obstructive Lung Disease
LLN	Lower Limit of Normal
PFTs	Pulmonary Function Tests
PRISm	Preserved Ratio Impaired Spirometry
RV	Residual Volume
SVC	Slow Vital Capacity
TLC	Total Lung Capacity
UFC	Unified Flow Chart
ULN	Upper Limit of Normal
VC	Vital Capacity
